# Prevalence and factors associated with chronic venous insufficiency, leg ulceration and deep-vein thrombosis among people who inject drugs in London, UK

**DOI:** 10.1111/dar.13389

**Published:** 2021-10-03

**Authors:** JASON DORAN, VIVIAN HOPE, TALEN WRIGHT, JENNY SCOTT, DANIEL CICCARONE, MAGDALENA HARRIS

**Affiliations:** 1 Faculty of Public Health and Policy, London School of Hygiene and Tropical Medicine, London, UK; 2 National Infection Service, Public Health England, London, UK; 3 Public Health Institute, Liverpool John Moores University, Liverpool, UK; 4 Division of Psychiatry, University College London, London, UK; 5 Department of Pharmacy and Pharmacology, University of Bath, Bath, UK; 6 Department of Family and Community Medicine, University of California, San Francisco, USA

**Keywords:** chronic venous insufficiency, deep vein thrombosis, harm reduction, leg ulcer, people who inject drug

## Abstract

**Introduction.:**

People who inject drugs (PWID) are vulnerable to a range of harms, including vascular conditions such as chronic venous insufficiency (CVI), leg ulcers and deep vein thrombosis (DVT). The extent of vascular conditions has rarely been studied, despite contributing to considerable illness and disability among PWID. We assess the prevalence and associations of vascular conditions in PWID in London, UK.

**Methods.:**

Survey data from the community-recruited Care and Prevent Study of PWID in London were analysed. Participants were asked about CVI and leg ulcers using pictorial questions, and if they had ever been diagnosed with DVT. Associations between vascular conditions and demographic/drug-use information were explored using univariate and multivariable logistic regression.

**Results.:**

Among participants (n = 455), the prevalence of CVI, leg ulcers and DVT was 13% (n = 57), 10% (n = 46) and 23% (n = 105), respectively. CVI and DVT were positively associated with injecting into the groin, while injecting into the leg was positively associated with leg ulcers and DVT. CVI was also associated with not cleaning injection sites and diagnosed hepatitis C virus, and DVT with hepatitis C virus.

**Discussion and Conclusion.:**

The prevalence of vascular problems among PWID in London is very high in comparison to the general population. These conditions are primarily associated with injection into the femoral vein. Use of these injection sites indicates peripheral venous access problems. There is a need to reinvigorate safe injection information provision in harm reduction services, with attention to reducing risk practices associated with venous damage and transitions to femoral injection.

## Introduction

There are an estimated 15 million people who inject drugs (PWID) worldwide [1], with the UK having one of the highest levels of injection drug use in Western Europe [2]. Illicit drug injection is associated with high levels of morbidity and premature mortality and increases susceptibility to a range of injecting-related harms, including blood-borne viral infections, such as hepatitis C (HCV) and human immunodeficiency virus (HIV), skin and soft tissue infections, overdoses and vascular damage [3,4].

Injecting drug use is a risk factor for venous damage, including deep vein thrombosis (DVT), chronic venous insufficiency (CVI) and leg ulceration. DVT, where occlusion of the vein occurs due to the formation of a thrombus on the vein wall, can cause irreversible damage, including peripheral oedema and post-thrombotic limb symptoms, such as ulcers and varicose eczema [5–7]. A study of people receiving treatment for opioid use in Middlesbrough, UK estimated a DVT prevalence of 14% [7], compared to approximately 0.1% in general adult populations [8,9], indicating that DVT is more common among PWID. DVT in PWID may be due to endothelial damage from injecting, potentially related to the overuse of acidifiers in drug preparation [10], as well high levels of coagulative factors as a result of injecting-related infections [11,12].

CVI, a consequence of venous damage in the lower extremities [13], has not been systematically studied among PWID, but is reported as common, with 87% of those examined at a treatment centre in the USA exhibiting clinical evidence of CVI [14]. Clinical manifestations of CVI include the appearance of varicose veins, oedema and darkened, dry skin on the lower extremities [14]. CVI and the ensuing vascular hypertension can give rise to the development of chronic leg ulceration [15–17]. Leg ulcers, defined as ‘breaks in the skin between the knee and the ankle, present for four weeks or more’ [18], appear to disproportionately impact PWID. A Scottish study, for example, reported a 15% prevalence of leg ulceration among PWID [19] in comparison with a 1–2% prevalence in the general population studies in high-income countries [20–22].

As well as posing a substantive cost to health-care systems [23], venous complications can be severely damaging to both physical and mental health. PWID with leg ulceration are frequently subject to chronic pain, mobility restrictions and also stigma and shame, including in relation to the management of open and odoriferous wounds [24,25]. Subsequently, this can act to restrict social mobility and employment options, compromise access to health care and exacerbate self-medication with illicit opioids for pain relief [13,25–27].

Harm reduction research, policy and practice have traditionally been oriented around the prevention and treatment of blood-borne viral infections and, more recently, skin and soft tissue infections [28]. Given the significant individual and social burden of injecting-related vascular issues, there is an urgent need to develop acceptable, accessible and effective preventative interventions. In order to implement such interventions, a greater understanding of the prevalence and risk factors for vascular issues among PWID is required, yet these have rarely been studied. In this study, we aimed to estimate the prevalence of vascular issues among PWID in London, UK and examine the associated factors, through the analysis of data from an in-depth cross-sectional survey exploring injecting practices and harms among PWID in London.

## Methods

### Data

Anonymised quantitative survey data from the mixed methods Care and Prevent Study (C&P) were used. Detailed methods and study rationale for C&P have previously been published [29]. The in-depth survey included questions surrounding injection-related comorbidities, drug-use history, injecting practices, sociodemographic characteristics and health-care access. Participants completed a detailed researcher-administered, computer-assisted survey.

### Ethical approval

Ethical approvals were obtained from the London Bridge Research Ethics Committee (17/LO/0872) and the London School of Hygiene and Tropical Medicine Observational Research Ethics Committee (12021). Written consent was obtained from all participants in the study.

### Study sample recruitment and eligibility

Participants were recruited from six treatment centres, homeless hostels and outreach services across London, UK. Eligibility to participate in the survey was restricted to those aged more than 18 years, who had ever injected psychoactive drugs and who were assessed as being able to provide informed consent. Participants were not approached directly for the study but were fully informed about the study through a detailed participant information sheet and researchers were present at the sites to provide further information on specified days. Interested participants then contacted the research team directly. Participants received a £10 voucher as reimbursement for their time. In total, 455 PWID completed the survey between October 2017 and March 2019.

### Study measurements

The C&P study questionnaire was carefully developed through extensive consultation with a panel of experts. The survey asked participants questions surrounding sociodemographic details; drug use history, injection preparation and administration practices (lifetime and previous 12 months); reuse and cleaning of injecting equipment (lifetime); and experience of skin and soft tissue infections, vascular conditions and other health conditions, including HIV and HCV. Questions pertaining to ever having CVI and leg ulceration were accompanied by pictures of typical condition presentation, to indicate their type and severity. Participants were also provided with a verbal description of symptoms in order to aid identification and nomination of severity, minimising misclassification bias. Participants were asked if they had ever been diagnosed by a health-care worker as having had DVT.

Participants answered detailed questions on reuse of injecting equipment, injecting hygiene practices, primary drugs injected, type and amount of acidifier used to prepare injection solutions, number of injections per day, week or month, body sites injected and number of times taken to achieve an injection. The latter question was included as it provides a strong indication of peripheral venous damage [30].

Participants who reported a lifetime history of CVI or leg ulcers were asked about the duration of the condition, how long it took them to seek care and if they had ever been hospitalised for that condition. Participants who reported a previous diagnosis of DVT were asked how long it took them to seek care prior to diagnosis. Measures pertaining to care seeking were informed by prior epidemiological injecting-related infection risk factor and hospitalisation research [31], which used five or more days as an indicator of health-care delay. We also specified 10 or more days to measure of the extent of delay, in recognition of the multiple barriers many PWID face in seeking care [4].

### Case definitions

The dependent variables in this study were a lifetime history of CVI, leg ulcers and diagnosed DVT. A lifetime history of CVI was defined as participants who reported ever having CVI based on pictures of the typical CVI presentation included in the survey. A lifetime history of leg ulcers was defined as participants who reported ever having a leg ulcer based on pictorial guidance included in the survey. Finally, a lifetime history of DVT was defined as participants who reported ever being diagnosed with DVT in the past.

### Statistical analysis

Data analysis was performed in Stata 16.1 (StataCorp LLC, College Station, TX, USA). Unless otherwise stated, all variables included in this analysis are reported across the participants’ history of injecting. Descriptive statistics, including means, medians and ranges, were used to present demographic and background characteristics of the entire sample and of those reporting a lifetime history of CVI, leg ulcers and diagnosed DVT. Univariate logistic regression was performed to investigate the associations between demographic and substance-use characteristics (independent variables) and lifetime history of CVI, leg ulcers and DVT (dependent variables), estimating unadjusted odds ratios and their 95% confidence intervals. Characteristics associated with vascular issues from the univariate analysis, where the *P*-value was <0.1, were included in the multivariable logistic regression model. Separate models for each of the three vascular issues were built including the likely confounding variables age and gender, *a priori*. A forward’s stepwise entry multivariable logistic regression was built to estimate adjusted odds ratios and their 95% confidence intervals. At each forward step, retention in the model was dependent either on the factor having the largest confounding effect or the strongest evidence of being an independent risk factor. Multivariable associations were deemed significant if *P*-values obtained by the Wald tests were <0.05.

## Results

### Prevalence of CVI, leg ulcers and DVT

Of the 455 participants, 13% (*n* = 57) reported lifetime history of CVI, 10% (*n* = 46) reported leg ulceration and 23% (*n* = 105) had been diagnosed with DVT (Table 1). Of those reporting CVI, 37% (*n* = 21) had lived with this for 5 or more years, 46% (*n* = 26) had received related medical care and 16% (*n* = 9) were hospitalised as a result. Of those reporting leg ulceration, 11% (*n* = 5) had lived with this for more than 5 years, 74% (*n* = 34) had received related medical care and 37% (*n* = 17) were hospitalised. The proportion of participants who reported taking 10 or more days to seek medical advice from first noticing symptoms of the condition was 28% (*n* = 16) in those with CVI and 20% (*n* = 9) in those with leg ulcers.

### Socio-demographic characteristics and unadjusted associations with venous issues

The mean age of participants was 46 years and 75% (*n* = 341) identified as male. More than half of participants (54%, *n* = 244) self-reported an HCV diagnosis and the majority (78%, *n* = 355) reported a lifetime history of street homelessness, with a mean duration of 4 years (Table 2). The percentage of participants who were ever homeless was 86%, 85% and 83% among those with a lifetime history of CVI, leg ulcers and DVT, respectively. Diagnosed HCV and increasing number of years homeless were crudely associated with all three conditions. In addition, increasing age in years was crudely associated with CVI, while female gender was protective against CVI (Table 2).

### Substance use characteristics and unadjusted associations with venous issues

As shown in Table 3, 42% (*n* = 192) of participants injected drugs for 15 or more years and 59.5% (*n* = 271) primarily injected either heroin, crack or heroin and crack combined in the previous 12 months. Table 3 shows 70% (*n* = 317) reported injecting more than 1 times per day, and the use of higher risk body sites for injection was common: 60.5% (*n* = 275) had ever injected into their leg, 41.5% (*n* = 189) had ever injected into their groin and 37.1% (*n* = 169) had ever injected into their neck. Twenty-five percent of participants (*n* = 113) typically took four or more attempts (skin punctures) to achieve an injection. Two-thirds (67%, *n* = 306) sometimes or always reused needles and almost one-quarter (22.6%, *n* = 103) never wiped the injection site prior to injecting. Crude associations showed that CVI was positively associated with injecting for more than 15 years; ever injecting into the leg, groin or neck; and never cleaning the injection site prior to injecting (Table 3). Leg ulcers were more likely in PWID who reported: injecting for more than 15 years; injecting heroin, crack or heroin and crack combined as a main drug in the previous 12 months; ever injecting into the leg, groin or neck; and taking more than four attempts to achieve an injection (Table 3). Finally, DVT was more likely in those who reported injecting for more than 15 years; injecting heroin, crack, or heroin and crack combined as a main drug in the previous 12 months; injecting more than once per day; and ever injecting into the leg, groin or neck (Table 3).

### Factors associated with CVI, leg ulcers and DVT: multivariable analysis results

Factors associated with CVI, leg ulcers and DVT among PWID in this study are shown in Table 4 as adjusted odds ratios. Following adjustment, ever injecting into the groin and leg was associated with DVT and ever injecting into the leg was associated with leg ulcers. CVI was also associated with never cleaning the injection site before injecting and diagnosed HCV. Finally, DVT was associated with diagnosed HCV (Table 4).

## Discussion

This study found a high prevalence of vascular damage among PWID in London. The prevalence of CVI, leg ulceration and DVT are markedly higher than the levels in the general adult population. DVT was the most common venous issue reported in this sample of PWID, with a lifetime prevalence of 23%. Given participants were only asked about diagnosed DVT, actual prevalence might be higher. In comparison, general population studies report DVT incidence of 1 per 10 000 in the adults aged under 40 years to 5–6 per 1000 in those aged 80 and over [8,9]. Leg ulceration was reported by 10% of our PWID participants, considerably higher than an approximately 1% lifetime prevalence reported among the general populations in high-income countries [20–22]. CVI prevalence, at 13% among our sample, also indicates disproportional impact on PWID, in comparison with a 6–9% prevalence among the few available studies pertaining to general populations in high-income countries [32]. This high prevalence of DVT, CVI and leg ulceration among our community sample of PWID accord with the few published estimates, primarily generated from samples of PWID receiving treatment for their drug use [33–35].

While the factors associated with each of the vascular conditions in this study varied, the strongest association for all was having ever injected into the femoral vein. Participants who reported ever using the femoral vein (42%) had almost 10, 7 and 2 times the odds of reporting DVT, CVI and leg ulcers, respectively, than those who never injected into the femoral vein. As damage to the femoral vein through injecting will likely impact returning blood flow in the area, this finding is expected. Transitions to using the femoral vein commonly occur due to difficult peripheral venous access—also indicated by our sample. We asked how many attempts (skin punctures) it took to achieve a typical injection, with 25% reporting four or more attempts. This, a clear marker of compromised venous access, is a risk for transition to the use of deeper veins such as the femoral or jugular veins, with potentially dangerous health consequences [10,36–38].

Compromised venous access and damage among PWID is likely due to scarring from repeated injections, including the possible overuse of acidifiers when injecting and/or compromised filtration practice [10,30]. Interventions to promote and maintain peripheral venous health are a key preventative measure for the vascular conditions described in this study. Harris *et al.*, for example, have detailed the role of structural and educational interventions in alerting the link between overuse of acidifier in injection preparation and venous damage [30]. Interventions orientated toward promoting venous care and safe injection preparation practice are also likely to resonate with PWID and provide a point of connection for other health and social care interventions [39].

As more broadly reported, difficulty in maintaining injecting related hygiene (such as cleaning sites prior to injection) can exacerbate the risk of bacterial infection and related complications [4]. People who are unstably housed, rough sleeping and/or have difficulty accessing fresh, clean water are likely to be most at risk of injection-related injuries and infections [40]. Therefore, it is crucial that interventions practically orientate toward providing sufficient equipment to support hygienic injection practice (such as clean water, hand wipes, alcohol wipes, injection preparation mats) as well as information on how to do so. Advocacy for the introduction of environments that support safe, hygienic injection practice is essential, particularly in countries that currently legislate against safe or supervised injection facilities.

We note high levels of hospitalisation for venous issues (16% for CVI and 37% for leg ulcers) in this sample of PWID and have published elsewhere on the association between the time taken to seek medical care, condition severity and hospitalisation [4]. Delaying or avoiding medical attention for venous conditions can be extremely dangerous. Untreated DVT, for example, can lead to potentially fatal pulmonary embolisms [41]. Although this particular analysis did not seek to identify specific barriers to the treatment of vascular issues, qualitative data from the Care and Prevent Study highlight the multiple barriers PWID face to health-care access, such as stigma, discrimination, poverty, limited geographical mobility, competing demands and fear of drug withdrawal if confined as an inpatient [42]. These and other barriers have been widely reported globally [43–45]. As we argue elsewhere [42], there is an urgent need for health-care system transformation so that PWID are able to engage early with a diversity of welcoming, flexible and accessible services.

Given the high prevalence of venous complications in PWID, screening for DVT, CVI and leg ulcers should be offered to PWID as part of general health-care provision. As self-care of injecting related injuries and infections is reportedly high among PWID [4], supporting best practice can reduce complication development. This could include provision of wound-care training, bandages and dressings. Although supporting self-care for venous ulcers is compromised by the need for specialist compression bandaging application, it might support wound healing for PWID unable to attend regular wound care appointments. As noted, PWID face multiple barriers to health-care access, particularly in relation to specialist services. It is therefore crucial that financial and policy support is provided for a range of innovative accessible interventions, such as in-reach wound care nurses at drug treatment and homeless hostel services and outreach wound care services for people living on the street [46].

### Strengths and limitations

This study measured wide-ranging injecting-related conditions; however, the inherent nature of a cross-sectional study meant we were unable to investigate temporal relationships between factors associated with the conditions and thus we cannot eliminate the possibility of reverse causality. Additionally, the nature of the study meant that only current injecting-related behaviour could be explored in detail, and past practices and risks may be different from current ones. However, in contrast to longitudinal studies which use health-care records, our study captures a community-based sample, who were not necessarily accessing health-care or drug treatment services and who have conditions that may not have been clinical recognised or recorded. Survey data may be subject to reporting bias as self-reporting of conditions, including those diagnosed, was used. Previous studies have, however, indicated that self-reporting of injecting-related infections is reliable among PWID [47]. In addition, this study used photographs to aid recall and self-diagnosis (see [48]). However, although some participants might have incorrectly reported their conditions, we believe such errors to be minimal. Answers relating to questions surrounding injection practices and hygiene may have been influenced by social desirability bias, though this is unavoidable and likely minimal.

The study aimed to be as representative as possible. Recruitment took place in community settings, thus aiming to engage a range of PWID—including those who might have difficulty accessing clinical services or hospitals. Although recruitment of participants was solely in London, comparative analysis demonstrates high comparability between characteristics of C&P participants and those of PWID recruited to a large national surveillance study in the UK [4]. This indicates the generalisability of PWID in the UK, with the acknowledgement of potential participant selection bias.

## Conclusion

The lifetime prevalence of vascular issues in PWID in London, UK is considerably higher than in the general population. Considering the associated health-care costs, distress and disability, interventions are urgently needed to reduce their occurrence, including removing the barriers to safe injecting practice, and improving access to care. Interventions which prevent vascular damage early in the causal pathway and help to promote safe injecting preparation and practice are likely to yield significant harm reduction benefits.

## Figures and Tables

**Table 1.** *Prevalence and treatment of CVI, leg ulcers and DVT among PWID (*n = *455)*

	CVI, % (*n*)	Leg ulcers, % (*n*)	DVT, % (*n*)
Prevalence	13 (57)	10 (46)	23 (105)
*Duration with venous issue*			
<5 years	63 (36)	89 (41)	a
5+ years	37 (21)	11 (5)	a
Received medical attention for the condition	46 (26)	74 (34)	a
Hospitalised with this condition	16 (9)	37 (17)	a
Took more than 10 days to seek medical advice for the condition	28 (16)	20 (9)	25 (26)

a Participants were not asked about this in relation to DVT. CVI, chronic venous insufficiency; DVT, deep vein thrombosis; PWID, people who inject drugs.

**Table 2.** *Demographic and health characteristics among people who inject drugs (*n = *455) stratified by CVI, leg ulcers and DVT*

Variable	Entire sample (*n* = 455)	CVI (*n* = 57)			Leg ulcers (*n* = 46)			DVT (*n* = 105)		
	*n* (%)/mean (IQR)	*n* (%)	Crude OR (95% CI)	*P*-value	*n* (%)	Crude OR (95% CI)	*P*-value	*n* (%)	Crude OR (95% CI)	*P*-value
*Age*										
Mean (IQR)	45.7 (44.9, 46.5)	48 (45.9, 50)	1.03 (1.01, 1.07)	0.04	46 (43.8, 48.7)	1.0 (0.97, 1.04)	0.65	46 (44.2, 47.6)	1.0 (0.98, 1.03)	0.77
*Gender*										
Male	341 (75)	50 (88)	1	0.01	35 (76)	1		83 (79)	1	0.26
Female	114 (25)	7 (12)	0.38 (0.14, 0.88)		11 (24)	0.93 (0.41, 1.97)	0.85	22 (21)	0.74 (0.42, 1.29)	
*Diagnosed hepatitis C*										
No	211 (46)	16 (28)	1	0.003	15 (33)	1		32 (30)	1	<0.001
Yes	244 (54)	41 (72)	2.46 (1.3, 4.9)		31 (67)	1.9 (0.96, 3.91)	0.05	73 (70)	2.39 (1.47, 3.93)	
*Number of years homeless*										
Mean (IQR)	4.1 (3.4, 4.7)	6.8 (3.9, 9.6)	1.1 (1.02, 1.1)	0.002	6.2 (3.0, 9.5)	1.05 (1.0, 1.1)	0.03	5.7 (4.1, 7.3)	2.23 (1.18, 4.43)	0.001

CI, confidence interval; CVI, chronic venous insufficiency; DVT, deep-vein thrombosis; IQR, interquartile range; OR, odds ratio.

**Table 3.** *Substance use characteristics among people who inject drugs (*n = *455) stratified by CVI, leg ulcers and DVT*

	Entire sample (*n* = 455)	CVI (*n* = 57)			Leg ulcers (*n* = 46)			DVT (*n* = 105)		
Variable	*n* (%)	*n* (%)	Crude OR (95% CI)	*P*-value	*n* (%)	Crude OR (95% CI)	*P*-value	*n* (%)	Crude OR (95% CI)	*P*-value
*Years injecting*										
<15 years	263 (58)	23 (40.3)	1	0.004	16 (35)	1	0.001	43 (41)	1	<0.001
15+ years	192 (42)	34 (59.7)	2.25 (1.23, 4.15)		30 (65)	2.86 (1.45, 5.79)		62 (59)	2.44 (1.53, 3.91)	
*Injected heroin, crack or heroin and crack combined in previous 12 months*										
No	184 (40.5)	23 (40.3)	1	0.99	10 (22)	1	0.006	31 (30)	1	0.001
Yes	271 (59.5)	34 (59.7)	1.0 (0.6, 1.9)		36 (78)	2.67 (1.25, 6.18)		74 (70)	1.85 (1.14, 3.07)	
*Typical injecting frequency*										
Inject less than once per day	138 (30)	15 (26.3)	1	0.48	17 (37)	1		23 (22)	1	0.03
Inject 1+ times per day	317 (70)	42 (73.7)	1.25 (0.7, 2.5)		29 (63)	0.72 (0.37, 1.45)	0.302	82 (78)	1.74 (1.02, 3.06)	
*Ever injected into the leg*										
No	180 (39.5)	12 (21.1)	1	0.002	5 (10.9)	1	<0.001	12 (11.4)	1	<0.001
Yes	275 (60.5)	45 (78.9)	2.74 (1.37, 5.86)		41 (89.1)	6.13 (2.35, 20.2)		93 (88.6)	7.15 (3.7, 14.8)	
*Ever injected into the groin*										
No	266 (58.5)	11 (19.3)	1	<0.001	15 (32.6)	1	<0.001	16 (15.2)	1	<0.001
Yes	189 (41.5)	46 (80.7)	7.46 (3.65, 16.4)		31 (67.4)	3.28 (1.65, 6.75)		89 (84.8)	13.9 (7.61, 26.5)	
*Ever injected into the neck*										
No	286 (62.9)	21 (36.8)	1	<0.001	19 (41.3)	1	0.001	40 (38.1)	1	<0.001
Yes	169 (37.1)	36 (63.2)	3.42 (1.85, 6.4)		27 (58.7)	2.67 (1.37, 5.26)		65 (61.9)	3.84 (2.38, 6.23)	
*Typical number of attempts to achieve injection*										
1–4 times	342 (75)	37 (65)	1	0.05	28 (61)	1	0.02	75 (71)	1	0.31
4+ times	113 (25)	20 (35)	1.8 (0.9, 3.3)		18 (39)	2.12 (1.1, 4.18)		30 (29)	1.29 (0.76, 2.15)	
*Typically wipe injection site before injecting*										
Always or sometimes	352 (77)	31 (54)	1	<0.001	31 (67)	1	0.088	78 (74)	1	0.39
Never	103 (23)	26 (46)	3.5 (1.9, 6.5)		15 (33)	1.77 (0.8, 3.54)		27 (26)	1.25 (0.72, 2.12)	
*Ever reuse needles/syringes*										
Never	149 (33)	13 (23)	1	0.08	12 (26)	1	0.3	26 (25)	1	0.05
Sometimes or always	306 (67)	44 (77)	1.8 (0.9, 3.7)		34 (74)	1.43 (0.7, 3.1)		79 (75)	1.65 (0.98, 2.82)	

CI, confidence interval; CVI, chronic venous insufficiency; DVT, deep-vein thrombosis; OR, odds ratio.

**Table 4.** *Factors associated with CVI, leg ulcers and DVT among people who inject drugs (*n = *455): multivariable regression results*

	CVI		Leg ulcers		DVT	
Variable	aOR (95% CI)	*P*-value	aOR (95% CI)	*P*-value	aOR (95% CI)	*P*-value
*Sex*						
Male	1	0.09	1	0.58	1 0.94 (0.49, 1.77)	0.84
Female	0.47 (0.19, 1.1)		1.23 (0.58, 2.62)			
Age, years	1.03 (0.99, 1.07)	0.08	0.99 (0.95, 1.03)	0.68	0.99 (0.97, 1.02)	0.93
*Diagnosed hepatitis C*						
No	1	0.03	^a^	–	1	
Yes	2.1 (1.10, 3.99)				1.83 (1.1, 3.13)	0.02
*Ever injected into the groin*						
No	1		1		1	
Yes	6.69 (3.28, 13.63)	<0.0001	1.96 (0.98, 3.93)	0.05	9.57 (5.2, 17.6)	<0.001
*Ever injected into the leg*						
No	^a^	–	1 3.92 (1.41, 10.86)	0.009	1 3.2 (1.57, 6.48)	<0.001
Yes						
*Typically wipe injection site before injecting*						
Always or sometimes	1		^a^	–	^a^	–
Never	3.03 (1.62, 5.68)	0.001				
*Years injecting*						
<15 years	^a^	–	1		1	
15+ years			1.95 (0.98, 3.93)	0.06	1.58 (0.98, 2.56)	0.07

a This variable was not included in the final model. aOR, adjusted odds ratio; CI, confidence interval; CVI, chronic venous insufficiency; DVT, deep vein thrombosis.
